# Urothelial Urinary Bladder Cancer Is Characterized by Stage-Dependent Aberrations in Metabolism of Bioactive Sphingolipids

**DOI:** 10.3390/ijms252211889

**Published:** 2024-11-05

**Authors:** Grzegorz Młynarczyk, Agnieszka Mikłosz, Adrian Chabowski, Marcin Baranowski

**Affiliations:** 1 Department of Urology, Medical University of Białystok, Skłodowskiej-Curie 24A, 15-276 Białystok, Poland; 2 Department of Physiology, Medical University of Bialystok, Mickiewicza 2c, 15-222 Białystok, Poland

**Keywords:** dihydrosphingosine-1-phosphate, S1PL, sphinganine, sphingosine-1-phosphate lyase, sphingosine-1-phosphate phosphatase, TCC, transitional cell carcinoma

## Abstract

Although dysregulated sphingolipid metabolism was observed in many malignant tumors, bladder cancer has not yet been examined in this regard. This study aims to investigate the metabolism of bioactive sphingolipids across different stages of urothelial urinary bladder cancer (UBC). Forty-eight patients with UBC were included in this study. The neoplasms were classified as either non-muscle-invasive (NMIBC, *n* = 24) or muscle-invasive (MIBC, *n* = 24). Samples of the healthy bladder tissue were taken from the patients who underwent radical cystectomy. The content of sphingolipids was measured using an HPLC method, and the mRNA expression of sphingolipid transporters and metabolizing enzymes was evaluated using RT-PCR. Compared to the healthy bladder tissue, the UBC, regardless of the stage, showed an elevated expression of SphK1, Spns2, and ABCC1. The changes in the level of bioactive sphingolipids were strongly stage-dependent. MIBC showed accumulation of sphingosine-1-phosphate (S1P) and ceramide, whereas the content of these sphingolipids in the NMIBC tumor was not different from that of healthy tissue. Moreover, MIBC, compared to NMIBC, was characterized by higher levels of sphingosine and dihydroceramide. We conclude that profound alterations in sphingolipid metabolism develop upon UBC transition from non-muscle-invasive to muscle-invasive. They include the accumulation of S1P, resulting from the increased availability of sphingosine generated from ceramide, which also builds up due to a further activation of its de novo synthesis. We hypothesize that the dysregulation of S1P metabolism leading to the accumulation of this tumor-promoting sphingolipid contributes to the progression of UBC.

## 1. Introduction

Sphingolipids are not only vital components of cell membranes, but also act as bioactive molecules essential in signal transduction and the regulation of numerous cellular processes, such as proliferation, growth, migration, differentiation, and apoptosis [1]. Ceramide, which stands out as the central molecule in sphingolipid metabolism, is mainly synthesized through two pathways—the hydrolysis of sphingomyelin via the action of sphingomyelinase and de novo synthesis. The de novo pathway begins with serine and palmitoyl-CoA condensation, catalyzed by serine palmitoyltransferase, producing 3-ketosphinganine, which is then rapidly converted to dihydrosphingosine. Subsequent steps include the acylation of dihydrosphingosine to dihydroceramide by ceramide synthase, followed by its conversion to ceramide via sphingolipid Δ4-desaturase (DEGS). Ceramide is primarily degraded to sphingosine by ceramidase [2].

**Figure 1:**
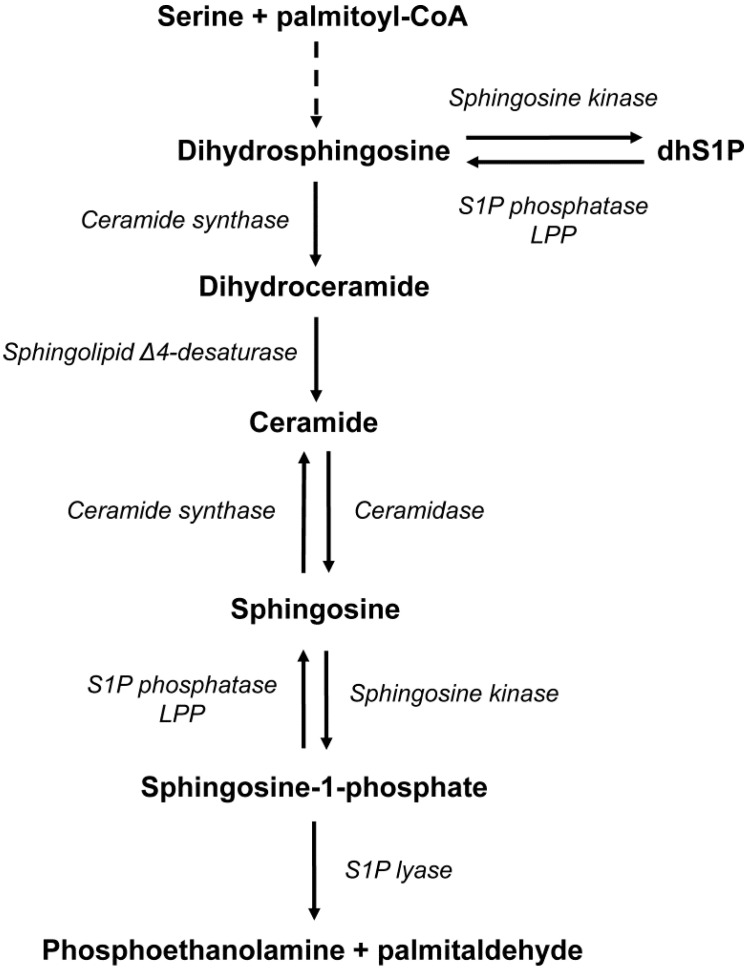
Schematic representation of the metabolism of sphingolipids investigated in the present study. Abbreviations: dhS1P—dihydrosphingosine-1-phosphate; LPP—lipid phosphate phosphohydrolase; S1P—sphingosine-1-phosphate. The dashed arrow indicates that some metabolites have been omitted.

Thereafter, sphingosine can either be reacylated into ceramide or converted into sphingosine-1-phosphate (S1P) by sphingosine kinase (SphK), which also phosphorylates dihydrosphingosine to form dihydrosphingosine-1-phosphate (dhS1P). These molecules are irreversibly broken down by sphingosine-1-phosphate lyase (S1PL) or dephosphorylated back to free sphingoid bases by S1P phosphatase (SPP) and nonspecific lipid phosphate phosphohydrolase (Figure 1) [3].

Although S1P and dhS1P may function as intracellular messengers, they exert most of their biological effects through membrane G-protein coupled receptors (S1PRs) after being exported to the extracellular space by ATP-binding cassette transporter C1 (ABCC1) or spinster homolog 2 (Spns2) [4].

Considerable evidence highlights the involvement of bioactive sphingolipids like S1P and ceramide in cancer development, progression, metastasis, and resistance to chemotherapy [5]. Ceramide is generally regarded as an anticancer agent due to its ability to arrest growth and induce apoptosis. The administration of ceramide analogs or pharmacological and genetic interventions to increase ceramide levels trigger caspase activation and apoptosis in cancer cells. Furthermore, sphingolipid metabolic pathways that promote ceramide production are critical for the therapeutic efficacy of radiation and many chemotherapeutic agents [6]. In contrast, S1P functions as a tumor promoter by supporting the cellular transformation, migration, proliferation, angiogenesis, and survival of cancer cells, while also inhibiting ceramide-induced apoptosis [7]. The modulation of S1P signaling is a promising avenue for cancer therapy, with SphK inhibitors and FTY720 (a functional S1PR antagonist) shown to inhibit cancer cell proliferation and tumor growth in both in vitro and in vivo models [8].

Aberrant sphingolipid metabolism was observed in numerous cancers, and specific bioactive sphingolipids were identified as biomarkers for disease severity and treatment response [9]. Urinary bladder cancer is the fourth most common carcinoma in men, and the most common malignancy of the urinary system [10]. However, the changes in sphingolipid metabolism associated with its development and progression have not been examined so far. Therefore, this study aims to investigate the metabolism of bioactive sphingolipids across different stages of urothelial urinary bladder cancer (UBC), the most common type of bladder carcinoma.

## 2. Results

### 2.1. Sphingolipid Content

**Figure 2:**
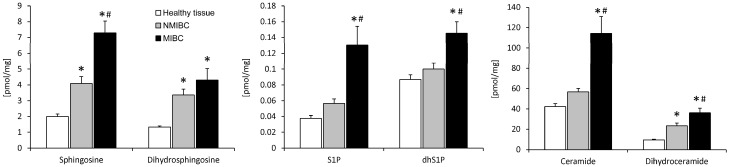
The content of bioactive sphingolipids in non-muscle-invasive (NMIBC) and muscle-invasive (MIBC) bladder cancer, and the adjacent healthy bladder tissue. The results are means ± SEM. *—*p* < 0.05 vs. the healthy bladder tissue; #—*p* < 0.05 vs. NMIBC. S1P—sphingosine-1-phosphate; dhS1P—dihydrosphingosine-1-phosphate.

Compared to the healthy bladder tissue, the non-muscle-invasive bladder cancer (NMIBC) was characterized by a ~2-fold higher content of sphingosine, dihydrosphingosine, and dihydroceramide, whereas the level of S1P, dhS1P, and ceramide was not significantly different (Figure 1). On the other hand, the muscle-invasive bladder cancer (MIBC) showed a much higher content of all examined sphingolipids than the healthy bladder tissue. The observed difference ranged from 1.7-fold for dhS1P to as much as ~3.5-fold for dihydroceramide, sphingosine, and S1P. Moreover, MIBC, compared to NMIBC, was characterized by a higher level of all examined sphingolipids, with the exception of dihydrosphingosine. The highest difference between the two investigated stages of UBC was observed for S1P and ceramide, which increased 2.3- and 2-fold, respectively (Figure 2).

### 2.2. Expression of mRNA

**Figure 3:**
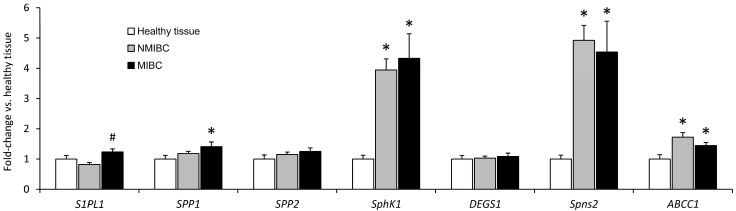
The mRNA levels of enzymes and transporters involved in sphingosine-1-phosphate metabolism in non-muscle-invasive (NMIBC) and muscle-invasive (MIBC) bladder cancer, and the adjacent healthy bladder tissue. The results are means ± SEM. *—*p* < 0.05 vs. the healthy bladder tissue; #—*p* < 0.05 vs. NMIBC. *ABCC1*—ATP-binding cassette transporter C1; *DEGS1*—sphingolipid Δ4-desaturase 1; *SphK1*—sphingosine kinase 1; *S1PL1*—sphingosine-1-phosphate lyase 1; *Spns2*—spinster homolog 2; *SPP*—sphingosine-1-phosphate phosphatase.

Compared to the healthy bladder tissue, the mRNA levels of *ABCC1* were higher in both NMIBC and MIBC (by 1.7- and 1.4-fold, respectively). A similar, albeit much stronger trend, was also observed for *SphK1* and *Spns2* expression (3.9- to 4.9-fold increase). The mRNA levels of the aforementioned genes were, however, not significantly different between the two investigated tumor stages. On the other hand, the expression of *SPP1* was increased only in MIBC (by 1.4-fold), and the mRNA level of *S1PL1* was significantly higher (by 1.5-fold) in MIBC neoplasms, compared to NMIBC. There were no statistically significant differences in the expression of *SPP2* and *DEGS1* (Figure 3).

## 3. Discussion

As already mentioned in the introduction, it has been firmly established that bioactive sphingolipids play a crucial role in cancer development, progression, and metastasis. However, information on sphingolipid metabolism in UBC is scarce, and most of the available data come from cell lines. In the present study, the sphingolipid profiles of UBC and the adjacent healthy bladder tissue were examined for the first time. In line with previous reports carried out on a broad range of tumor types [9], we found that UBC is characterized by major stage-dependent aberrations in the metabolism of bioactive sphingolipids.

Compared to the healthy bladder tissue, MIBC showed a striking accumulation of S1P. The same effect was observed for sphingosine, which, together with a marked increase in the expression of *SphK1*, indicates that the augmented rate of S1P synthesis was responsible for its accumulation. This conclusion is further supported by the fact that mRNA levels of S1P-degrading enzymes were not reduced in MIBC. In fact, the expression of *S1PL1* and *SPP1* was slightly increased, which likely represented a compensatory response for the accumulation of S1P. It should be noted that this effect was not observed in NMIBC, where S1P content was not elevated. Our observation of increased S1P levels in MIBC is in accordance with other studies conducted on several types of solid tumors, including endometrial carcinoma, glioblastoma, clear cell renal cell carcinoma, and breast cancer [11,12]. Although there are no literature data on the content of S1P in UBC, several reports found an increased expression of *SphK1* in bladder cancer compared with this in healthy adjacent tissue [13,14,15], which is in line with the results of our study.

In vitro experiments provided strong evidence for the important role of the SphK1/S1P axis in the progression and chemoresistance of bladder cancer. Wollny et al. [16] reported that exogenous S1P stimulated the proliferation of human urinary bladder cancer cells in a dose-dependent manner. Moreover, the silencing of SphK1 expression was found to prevent the hypoxia-induced increase in their invasion ability [15], whereas the overexpression of this enzyme promoted cell proliferation and reduced the apoptotic rate [13]. It was also reported that the overexpression of SphK1 alleviated the inhibitory effect of miR-613 and miR-125b-5p on the proliferation, migration, and invasion of bladder cancer cells [17,18]. The contribution of SphK1 to chemoresistance in bladder cancer cells was recently confirmed by Qin et al. [13].

As already mentioned in the introduction, S1P exerts most of its biological effects extracellularly, after being exported by ABCC1 and Spns2. We have shown for the first time that the expression of both genes was higher in UBC compared to the healthy bladder tissue. However, this effect was much stronger for the latter transporter, suggesting the important role of Spns2 in UBC. Similarly to our study, the expression of Spns2 was found to be increased in some neoplasms including colorectal cancer and clear cell renal cell carcinoma [12,19].

There is an increasing body of evidence stating that Spns2 promotes cancer development, cell survival, and metastasis, mainly via increasing the extracellular S1P concentration, which results in the activation of S1PRs [20]. Several studies found an elevated expression of S1PR_1_ in bladder cancer, especially in high-grade tumors [21,22,23,24]. A high S1PR_1_ expression in tumors was also associated with a poor clinical outcome [23,24].

Our results, as well as those of other authors, showed that UBC is characterized by the elevated expression of a complete set of genes required for the synthesis, export, and extracellular action of S1P. Nevertheless, as found in the present study, S1P accumulates only in the muscle-invasive tumor, which strongly suggests that this bioactive sphingolipid plays an important role in the progression of UBC. This conclusion is also supported by the fact that the S1P content in high-motility RT112 bladder cancer cells was found to be 3-fold higher than the one in low-motility RT4 cells [25].

Another stage-dependent difference in sphingolipid metabolism observed in our study was the content of ceramide. In NMIBC, it was not statistically different from the healthy bladder tissue, whereas in MIBC, it was markedly elevated. Importantly, the accumulation of ceramide was accompanied by a further increase in the level of dihydrosphingosine and dihydroceramide. It is, therefore, most likely that this effect resulted from the augmented rate of ceramide de novo synthesis. The activation of this pathway has previously been reported for many types of neoplasms [11,12,26,27]. Interestingly, the accumulation of ceramide in MIBC was associated with a marked increase in the content of sphingosine. Augmented ceramide degradation likely contributed to the S1P accumulation observed in MIBC via the generation of the substrate for its synthesis. This notion is also supported by a strong positive correlation between the content of ceramide and S1P in MIBC (r = 0.68, *p* < 0.001) but not NMIBC (r = 0.31, *p* = 0.16).

## 4. Materials and Methods

### 4.1. Study Design and Participants

The material was collected during surgical procedures performed at the Department of Urology of the Medical University of Białystok. In total, 48 patients (8 women and 40 men) with a urinary bladder tumor diagnosed during USG, CT, or cystoscopy were included in the study. The neoplasms were classified as either non-muscle-invasive (NMIBC) (*n* = 24) or muscle-invasive (MIBC) (*n* = 24) bladder cancer [28]. Patients from the NMIBC group underwent transurethral resection of the bladder tumor, whereas those from the MIBC group were subjected to radical cystectomy. Only the neoplasms with a histopathologically confirmed diagnosis (by two independent pathologists) of urothelial urinary bladder cancer were included in the study. All tumors from the NMIBC group were classified as stage pTa—non-invasive papillary carcinoma—whereas all neoplasms from the MIBC group were diagnosed as stage pT2—muscle-invasive tumor. We qualified only subjects with a pathological diagnosis of pTa and pT2 due to the priority to obtain the most uniform groups of patients. The main anthropometric and clinical characteristics of the subjects studied are summarized in Table 1.

As soon as the urinary bladder was removed or transurethral bladder tumor resection was performed, samples were collected from a macroscopically visible proliferative lesion. The comparative material was taken from the patients who underwent radical cystectomy and consisted of samples of the urinary bladder taken from the pole of the organ opposite the tumor. These fragments macroscopically showed no neoplastic or inflammatory features. Additionally, during the assessment of a cancerous organ, pathologists evaluated both the nodular lesion and the surrounding tissues in order to exclude macroscopically invisible tumor foci. If cancer cells were identified in the region where the healthy tissue was sampled, the patient was excluded from the study. The obtained samples were washed with 0.9% NaCl solution, portioned, weighed, and stored at −80 °C until the analysis.

### 4.2. Sphingolipid Analysis

The content of S1P, dhS1P, sphingosine, dihydrosphingosine, ceramide, and dihydroceramide was determined as described previously in detail [29]. Briefly, lipids were extracted from samples in the presence of internal standards (C17-sphingosine and C17-S1P, Avanti Polar Lipids, Alabaster, AL). An aliquot of the lipid extract was transferred to a fresh tube with pre-added N-palmitoyl-D-erythro-sphingosine (C17 base) (Avanti Polar Lipids) as an internal standard, and then subjected to alkaline hydrolysis to deacylate ceramide and dihydroceramide to free sphingosine and dihydrosphingosine, respectively. The amount of S1P and dhS1P was determined indirectly after dephosphorylation to sphingosine and dihydrosphingosine, respectively, with the use of alkaline phosphatase (bovine intestinal mucosa, Sigma, St. Luis, MO, USA). Free sphingosine and dihydrosphingosine, dephosphorylated sphingoid bases, as well as sphingosine and dihydrosphingosine released from ceramide and dihydroceramide, respectively, were then converted to their o-phthalaldehyde derivatives and analyzed using a UPLC system (Nexera, Shimadzu Corp., Kioto, Japan) equipped with a fluorescence detector (RF-20Axs) and a C18 reversed-phase column (Reproshell ODS-1, 2.7 μm, 125 × 3 mm, Dr Maisch, Ammerbuch, Germany). An isocratic eluent composition of acetonitrile:water (84:16, *v*/*v*) and a flow rate of 0.4 mL/min were used. The column temperature was maintained at 30 °C.

### 4.3. Real-Time PCR

Total RNA was isolated from the samples using the NucleoSpin RNA Plus Kit (Macherey Nagel GmbH & Co. KG, Duren, Germany) according to the manufacturer’s instructions. Spectrophotometric measurements were made to assess the quantity and quality of the extracted RNA (Synergy H1 Hybrid Reader, BioTek Instruments, Winooski, VT). Reverse transcription was performed using the EvoScript universal cDNA master kit (Roche Molecular Systems, Boston, MA, USA). Real-time PCR was performed using the LightCycler 96 System with FastStart essential DNA green master (Roche Molecular Systems) as the detection dye. Specific primers were designed using the Beacon Designer Software v. 8.20 (Premier Biosoft, Palo Alto, CA, USA); their sequences are listed in Table 2. The following reaction parameters were applied: initial denaturation at 95 °C for 10 min, followed by 45 cycles of 15s denaturation at 94 °C, 15 s annealing at 54 °C for *S1PL1*, 60 °C for *Spns2* and *SPP2*, 59 °C for *ABCC1* and ribosomal protein L13A (*RPL13A*), 58 °C for *SPP1* and *SphK1*, 61 °C for *DEGS1*, and 15 s extension at 72 °C. Melting curve analysis was performed at the end of each reaction to verify PCR product specificity. All samples were assayed in duplicate. Six reference genes (glyceraldehyde 3-phosphate dehydrogenase, RPL13A, β-actin, tumor protein translationally controlled 1, ribosomal protein S13, and ribosomal protein S23), which were previously found to be suitable for real-time PCR data normalization in UBC [30], were tested. In our cohort, *RPL13A* was identified as the reference gene with the most stable expression. The results were normalized against this housekeeper gene, and were calculated according to the Pfaffl method [31].

### 4.4. Statistical Analysis

All data are presented as means ± SEM. Statistical comparisons were made by using one-way ANOVA followed by the Newman–Keuls post hoc test. *p* < 0.05 was considered statistically significant.

## 5. Conclusions

In summary, compared to the healthy bladder tissue, UBC, regardless of the stage, was characterized by an elevated expression of *SphK1*, *Spns2*, and *ABCC1*. The changes in the level of bioactive sphingolipids were, on the other hand, strongly stage-dependent. MIBC showed a striking accumulation of S1P and ceramide, whereas the content of these sphingolipids in NMIBC was not different from that of the adjacent healthy tissue. Moreover, MIBC, compared to NMIBC, was characterized by higher levels of sphingosine and dihydroceramide. We conclude that UBC is characterized by the elevated expression of a whole set of genes involved in the synthesis and export of S1P. In addition, profound alterations in sphingolipid metabolism develop upon tumor transition from non-muscle-invasive to muscle-invasive bladder cancer. They include the accumulation of S1P resulting from the increased availability of sphingosine generated from ceramide, which also builds up due to a further activation of its de novo synthesis. We hypothesize that the dysregulation of S1P metabolism leading to the accumulation of this tumor-promoting sphingolipid contributes to the progression of UBC.

## Figures and Tables

**Table 1:** Anthropometric and clinical characteristics of the subjects.

Tumor Stage	NMIBC (*n* = 24)	MIBC (*n* = 24)
Sex (males/females)	19/5	21/3
Age (years)	68.8 ± 2.1	69.7 ± 1.8
BMI (kg/m^2^)	28.5 ± 0.9	27.9 ± 0.6
Distant metastasis (%)	0	0
Nodal invasion (%)	0	0
Hyperthyroidism (%)	8.3	4.2
Arterial hypertension (%)	62.5	29.2
Hypercholesterolemia (%)	29	50
Hypertriglyceridemia (%)	29	50
Type 2 diabetes (%)	8.3	4.2
Coronary disease (%)	0	4.2
Atrial fibrillation (%)	16.7	0

Data are presented as means ± SEM, unless stated otherwise. BMI—body mass index; MIBC—muscle-invasive bladder cancer; NMIBC—non-muscle-invasive bladder cancer.

**Table 2:** Forward and reverse primers used in real-time PCR.

Gene	GenBank Accession No.	Forward Primer	Reverse Primer
*SphK1*	NM_001142601.1	5′-CCTACTTGGTATATGTGCC-3′	5′-TCGCTAACCATCAATTCC-3′
*S1PL1*	NM_003901.3	5′-CAGAGTCAAGCCAAGGAT-3′	5′-GTATGGAGCAGCAATAAGC-3′
*SPP1*	NM_030791.4	5′-CATCATCATCGGGCTTCATT-3′	5′-TAGTATCTCGGCTGTGTCTC-3′
*SPP2*	NM_001320834.1	5′-TACGGCTGTCTTGCTACTACC-3′	5′-ACCACGGACGACCAATGA-3′
*ABCC1*	NM_004996.4	5′-CGGTGAAGGTTGTGTACTC-3′	5′-CCTCCTCATTCGCATCCA-3′
*Spns2*	NM_001124758.3	5′-ACACTGTCTCACTGTCTCG-3′	5′-TGATGCCAGCTTGTCAGA-3′
*RPL13A*	NM_012423.4	5′-CTATGACCAATAGGAAGAGCAACC-3′	5′-GCAGAGTATATGACCAGGTGGAA-3′
*DEGS1*	NM_001321541.2	5′-CAAACATTCCAAACCAGCGAT-3′	5′-GCAGTTGCATTAACCACTCAA-3′

*ABCC1*—ATP-binding cassette transporter C1; *DEGS1*—sphingolipid Δ4-desaturase 1; *RPL13A*—ribosomal protein L13A; *SphK1*—sphingosine kinase 1; *S1PL1*—sphingosine-1-phosphate lyase 1; *Spns2*—spinster homolog 2; *SPP*—sphingosine-1-phosphate phosphatase.

## Data Availability

The data that support the findings of this study are available from the corresponding author upon reasonable request.
